# Cannabinoid Receptor Agonist Inhibits Atrial Electrical Remodeling in a Tachypaced *Ex Vivo* Rat Model

**DOI:** 10.3389/fphar.2021.642398

**Published:** 2021-04-22

**Authors:** Danielle I. Lee, Michael Murninkas, Sigal Elyagon, Yoram Etzion, Hope D. Anderson

**Affiliations:** ^1^ College of Pharmacy, Rady Faculty of Health Sciences, University of Manitoba, Winnipeg, MB, Canada; ^2^ Canadian Centre for Agri-Food Research in Health and Medicine (CCARM), Albrechtsen Research Centre, St Boniface Hospital, Winnipeg, MB, Canada; ^3^ Cardiac Arrhythmia Research Laboratory, Department of Physiology and Cell Biology, Faculty of Health Sciences, Ben-Gurion University of the Negev, Beer-Sheva, Israel; ^4^ Regenerative Medicine and Stem Cell Research Center, Ben-Gurion University of the Negev, Beer-Sheva, Israel

**Keywords:** atrial fibillation, atrial refractoriness, cannabinoid 1 and cannabinoid 2 receptor agonists, atrial remodeling, Cannabinoid

## Abstract

**Introduction:** Atrial fibrillation (AF) leads to rate-dependent atrial changes collectively defined as atrial remodelling (AR). Shortening of the atrial effective refractory period (AERP) and decreased conduction velocity are among the hallmarks of AR. Pharmacological strategies to inhibit AR, thereby reducing the self-perpetual nature of AF, are of great clinical value. Cannabinoid receptor (CBR) ligands may exert cardioprotective effects; CB13, a dual CBR agonist with limited brain penetration, protects cardiomyocytes from mitochondrial dysfunction induced by endothelin-1. Here, we examined the effects of CB13 on normal physiology of the rat heart and development of tachypacing-induced AR.

**Methods:** Rat hearts were perfused in a Langendorff set-up with CB13 (1 µM) or vehicle. Hemodynamic properties of non-paced hearts were examined conventionally. In a different set of hearts, programmed stimulation protocol was performed before and after atrial tachypacing for 90 min using a mini-hook platinum quadrupole electrode inserted on the right atrium. Atrial samples were further assessed by western blot analysis.

**Results:** CB13 had no effects on basal hemodynamic properties. However, the compound inhibited tachypacing-induced shortening of the AERP. Protein expression of PGC1α was significantly increased by CB13 compared to vehicle in paced and non-paced hearts. Phosphorylation of AMPKα at residue threonine 172 was increased suggesting upregulation of mitochondrial biogenesis. Connexin43 was downregulated by tachypacing. This effect was diminished in the presence of CB13.

**Conclusion:** Our findings support the notion that peripheral activation of CBR may be a new treatment strategy to prevent AR in patients suffering from AF, and therefore warrants further study.

## Introduction

Atrial fibrillation (AF) is a common, recalcitrant-to-treatment arrhythmia associated with severe complications including thromboembolic events, heart failure progression, reduced quality of life and increased mortality (Wolf et al., 1998; Nattel and Harada, 2014; Lippi et al., 2020). The prevalence of AF doubles every decade of life and is associated with multiple comorbidities including, but not limited to, structural heart disease, arterial hypertension, obesity, diabetes and sleep apnea (Ehrlich et al., 2002; Heijman et al., 2016; Anter et al., 2017; Hayashi et al., 2017; Nattel and Dobrev, 2017). Current approaches for rhythm control in AF patients are far from optimal. Ablation therapies are invasive and limited by cost, complexity, potential life-threatening complications and uncertain long-term outcome (Ramirez et al., 2020). Available drugs are only modestly effective, and some have unfavorable side effects including predisposition to life-threatening arrhythmias (Dobrev and Nattel, 2010).

AF has a self-perpetuating and self-exacerbating nature; thus, AF patients are progressively more prone to recurrence and persistence of the arrhythmia. The susceptibility of atrial tissue to AF (i.e. AF substrate) is developed through structural and electrical remodeling in ways that are still being elucidated (Li nd Brundel, 2020; Nattel et al., 2020). Increasing evidence indicates that cardiac dysmetabolism plays a central role in the pathophysiology of AF-related atrial remodelling as well as in the AF substrate of diabetic/pre-diabetic patients (Lee et al., 2020). In fact, dysfunction of adenosine monophosphate-activated protein kinase (AMPK), which is a major regulator of cardiac metabolism, can lead to cellular abnormalities and increase AF substrate (Harada et al., 2012; Qiu et al., 2016a).

During cardiovascular distress such as ischemia-reperfusion injury, the endocannabinoid system (ECS) is activated and reportedly produces beneficial effects (Wagner et al., 2001; Montecucco and Di Marzo, 2012). The ECS is composed of cannabinoid receptors (CBR), endocannabinoid (eCB) ligands, and enzymes to biosynthesize, degrade and transport eCBs (Moreno et al., 2019). All components of the ECS are present in the heart. (Galiegue et al., 1995; Schmid et al., 2000; Bonz et al., 2003). Furthermore, in response to endogenous and external stimuli, eCBs are produced and interact with CBRs to exert regulatory actions toward the maintenance of internal homeostasis through adaptive cellular modification (Moreno et al., 2019). Therefore, exogenous cannabinoid interactions with the cardiovascular ECS and CBRs may elicit beneficial effects to modulate complications of cardiomyopathies, such as arrythmias (Montecucco and Di Marzo, 2012).

CBRs are G protein-coupled receptors of which there are two types. CBR type 1 (CB1R) predominates in the central nervous system, and is the key signaling effector of any CB-dependent psychoactive actions (Matsuda et al., 1990; Pertwee et al., 2010). In contrast, ligand activation of CBR type 2 (CB2R) typically leads to immunomodulation such as immune cell migration and cytokine release (Munro et al., 1993). Agonists that bind to CB2R lack the psychoactive effects elicited by CB1R agonists in the brain (Munro et al., 1993; Pertwee et al., 2010). CB1R and CB2R are also found in the periphery and regulate processes in organs such as the gastrointestinal tract, lungs, skin, bone, heart and liver (Gebremedhin et al., 1999; Liu et al., 2000; Bonz et al., 2003; Bouchard et al., 2003).

Exogenous cannabinoids, whether plant-derived or synthetic, have a wide range of physiological effects through interaction with the ECS. While eCB actions are presumably limited to the cellular site in which they are synthesized and released, the delivery of exogenous cannabinoids into the body results in excess compound concentrations that are more widely distributed (Moreno et al., 2019). Thus, exogenous cannabinoid effects are much more prolonged compared to eCBs (Moreno et al., 2019). Synthetic cannabinoids, originally developed to act as pharmacological probes of the ECS, are derivatives of phytocannabinoids and eCBs (Le Boisselier et al., 2017). Hundreds of varieties of new and novel synthetic cannabinoids exist, as minor modifications to the chemical structure alter the affinity and selectivity to CBRs (Le Boisselier et al., 2017).

Using CB13, a CB1R and CB2R dual agonist that does not cross the blood-brain barrier, we previously reported antihypertrophic effects and attenuated mitochondrial dysfunction in cardiomyocytes via activation of AMPK signaling pathways (Lu et al., 2014; Lu et al., 2020). We hypothesize that the beneficial effects of CB13 may be relevant to stressed atria as well. Here, we used a Langendorff-perfused rat heart to assess the effects of CB13 on basic physiology of the heart as well as on the atrial electrophysiology following acute atrial tachypacing to mimic an atrial tachyarrhythmia. Our findings support the notion that the beneficial effects of CB13 are indeed relevant in the stressed atria and may serve as new therapeutic strategy to attenuate AF-related atrial remodeling.

## Materials and Methods

### Animals

Adult male Sprague-Dawley rats (*n* = 26, 200–250 g) obtained from Envigo Laboratories (Jerusalem, Israel) were used in the study. Experiments were approved by the institutional ethics committee of Ben-Gurion University of the Negev, Israel (Protocol No. IL-05-09-2018A) and were carried out in strict accordance with the Guide for the Care and Use of Laboratory Animals of the National Institutes of Health. The animals were kept under standardized conditions: 12:12 light:dark cycles at 20–24°C and 30–70% relative humidity. Animals were free-fed autoclaved rodent chow and had free access to reverse osmosis-filtered water. Hearts were excised from the animals under deep pentobarbital anesthesia.

### Isolated Perfused Heart Preparation and Basic Physiological Measurements

**FIGURE 1:**
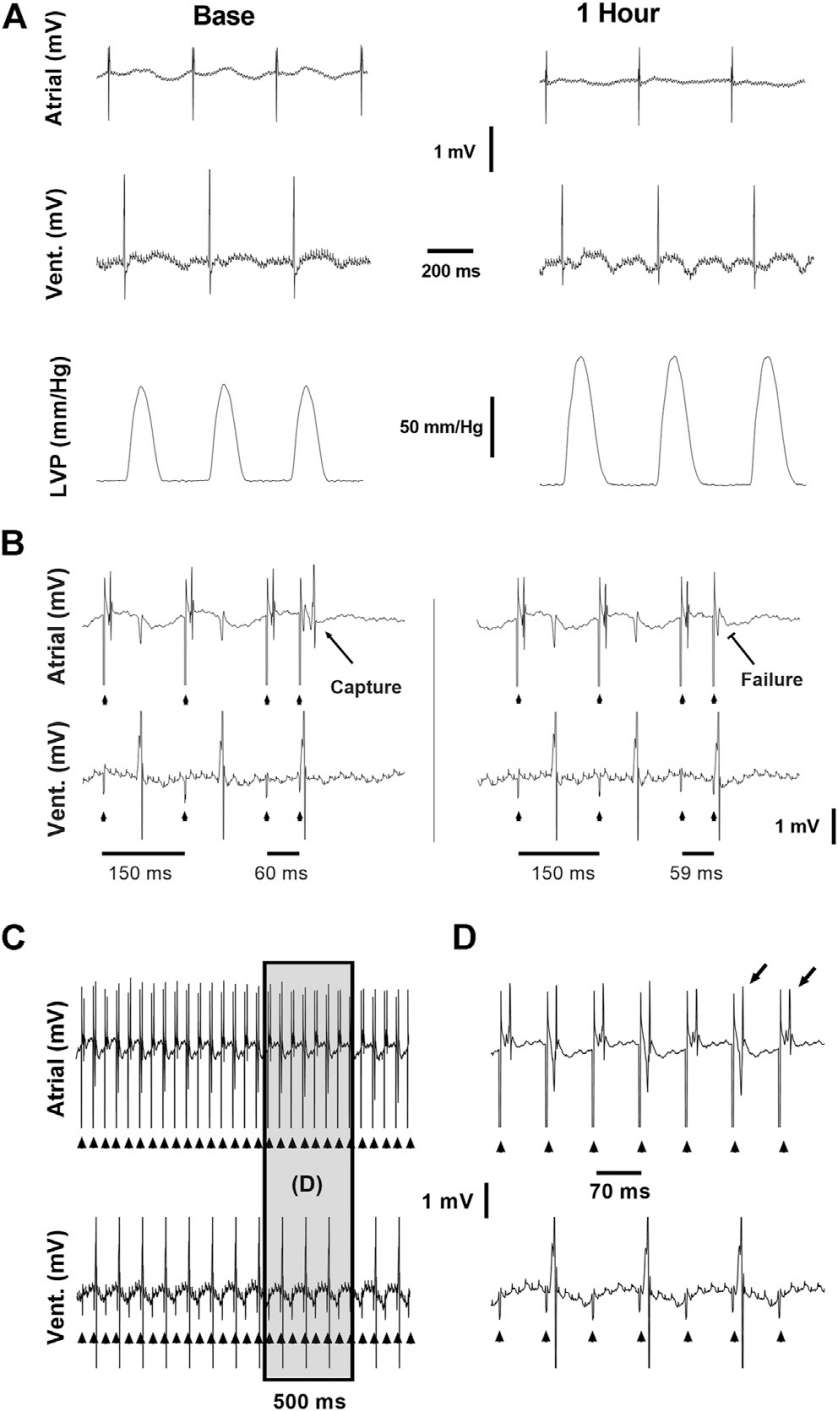
Example recordings from the *ex vivo* experimental setup. **(A)** Baseline **(left)** and 1 h **(right)** recordings demonstrating the atrial and ventricular electrical signals and LVP signal used to evaluate the effects of CB-13 under standard conditions without pacing. **(B)** Conventional S1S2 programmed stimulation protocol applied using a quadripolar electrode attached to the right atrium. Left, Atrial capture following S1S2 of 60 ms. Right, Failure of atrial capture following S1S2 of 59 ms. **(C)** Continuous assessment of atrial and ventricular signals during atrial tachypacing study. **(D)** Enlarged segment of the recording in C demonstrating the clear and precise ability to determine 1:1 atrial capture during the tachypacing protocol used in this study. Note beat-to-beat oscillation in the shape of the atrial signal (alternans) often observed when the fast pacing cycle length was reaching the minimal value that was able to maintain 1:1 atrial capture.

Isolated heart experiments were performed as previously described (Mor et al., 2013) with slight modifications as detailed below. Briefly, following intraperitoneal (IP) injection of heparin (1,000 units), each rat was anesthetized with pentobarbital (IP; 60 mg/kg) and the heart was excised into cold, oxygenated Tyrode's buffer (mM: 140 NaCl, 5.4 KCl, 0.5 MgCl_2_, 2.5 CaCl_2_, 0.39 NaH_2_PO_4_, 10 HEPES, and 11 glucose, pH 7.4). The aorta was cannulated and perfusion was initiated with oxygenated, pre-heated (37^o^C) Tyrode's solution. Perfusion rate was adjusted to maintain a constant coronary perfusion pressure of ∼80 mmHg. To obtain hemodynamic measurements, the left atrium was excised and a fluid-filled latex balloon was inserted into the left ventricle (LV) cavity through the mitral valve. The balloon was then inflated to an end-diastolic pressure of ∼5 mmHg. Electrophysiological signals from the right atria (RA) and LV were recorded using miniature-bipolar hook electrodes (Etzion et al., 2008). Coronary perfusion pressure and LV pressure were recorded by a pressure amplifier (ETH-256C amplifier and PB-100 probes, iWorx, NH, United States). Electrical signals were filtered (1–2 KHz) and recorded by two voltage amplifiers (Model 3000, A-M Systems, Carlsborg, WA, United States). Signals were interfaced with a PC using an A/D converter (PCI-6024E, National Instruments, Austin, TX, United States) and a custom-designed program developed with LabView programming language (National Instruments, Austin, TX, United States) to control signal acquisition, data saving and off-line analysis. Basic physiological measurements included spontaneous beating rate, atrioventricular (AV) interval, LV developed pressure, +dP/dt and −dP/dt. Parameters were recorded following a 20 min recovery period (Figure 1A). Thereafter, CB13 or vehicle were applied and measurements were repeated every 15 min for 1 h.

### Atrial Electrophysiology and Atrial Tachypacing Experiments

For atrial electrophysiology experiments, hearts were perfused as described above but a balloon was not inserted to the LV cavity and both atria remained intact. A custom-designed quadripolar-electrode was inserted on the RA for simultaneous pacing and recording (Murninkas et al., 2021). The electrode contained two pairs of platinum-iridium poles (one for pacing and one for recordings) with an inter-pair interval of 4 mm. The electrode was fixed to the atria by two delicate stainless steel pins and RA pacing and recording were verified. Hearts were continuously exposed to CB13 or vehicle throughout the experiments. A programmed S1S2 stimulation protocol was performed using a double threshold intensity, to measure atrial effective refractory period (AERP). The protocol consisted of 10 S1-S1 intervals of 150 ms followed by an S1-S2 interval that was reduced by 1 ms each time until atrial capture failed three consecutive times (Figure 1B). Conduction time was measured during constant atrial pacing at 150-ms CL as the interval between the end of the stimulus applied to one side of the electrode and the first peak of the atrial signal that was recorded in the other side (Murninkas et al., 2021). In addition to AERP and CT measurements, burst pacing was applied to the RA to evaluate for possible arrythmia induction. This protocol included 10 consecutive 20 s bursts at a cycle length of 20 ms. Following baseline measurements, the RA was continuously tachypaced for 90 min at double diastolic threshold. Tachypacing cycle length was adjusted dynamically and maintained 5 ms above the minimal cycle length in which 1:1 atrial capture was observed (Figure 1C). Following tachypacing, AERP and arrythmia induction protocols were repeated and the LA and a sample of the LV were snap frozen in liquid nitrogen for further biochemical analysis.

### Pharmacological Treatments

CB13 (1-naphthalenyl [4-(pentyloxy)-1-naphthalenyl]methanone) was from Cayman Chemical (Ann Arbor, Michigan). Tyrode’s buffer was supplemented with vehicle (0.1% v/v DMSO) or CB13 (1 μM, 0.1% v/v DMSO). Treatments remained in buffer for the remainder of the experiment. The concentration of CB13 was based on our previous findings in cardiomyocytes *in vitro* (Lu et al., 2014; Lu et al., 2020).

### Western Blotting

Lysates were prepared in radioimmune precipitation assay (RIPA) buffer with phosphatase and protease inhibitors using a bead mill homogenizer and clarified by centrifugation. Total protein concentration was determined by BCA assay (Thermo Fisher Scientific, Massachusetts, United States). Equal amounts of protein were loaded on Mini-PROTEAN TGX stain-free gels (Bio-Rad, California, United States) and transferred to PVDF membrane (Bio-Rad). Membranes were blocked using Tris-buffered saline with 0.1% Tween-20 (TBST) and 5% bovine serum albumin (BSA; Sigma Aldrich, Oakville, Ontario). Primary antibodies Phospho-AMPKα (Thr172) (1:1,000, cat. 2,535), AMPKα (1:1,000, cat. 2,603), phospho-LKB1 (1:1,000, cat. 3,482), LKB1 (1:1,000, cat. 3,047) were from Cell Signaling Technology (Danvers, Massachusetts) and PGC-1α (1:1,000, cat. ab72230), connexin 43 (1:7,500, cat. ab11370), CB1R (1:500, cat. ab23703), and CB2R (1:5,000, cat. ab45942) antibodies were from Abcam (Toronto, Ontario). Primary antibodies were incubated overnight at 4°C. Affinity-purified horseradish peroxidase-linked secondary antibodies were from Cell Signaling. As applicable, membranes were stripped and reprobed. Membranes were normalized to total protein concentration (TPN) using Bio-Rad stain-free technology, measured using a Bio-Rad Chemidoc™ MP Imaging system and analyzed using Image Lab Software (Mississauga, Ontario) to account for variation in loading among lanes.

### Statistical Analysis

Data are expressed as mean ± SEM. GraphPad Prism 9 software was used for analysis. Normality assumptions were tested using the Shapiro-Wilk test for normality. For analysis between more than two groups: when 1 or more groups did not pass the normality test, a Kruskal-Wallis test followed by Dunn’s *post hoc* for multiple comparisons was performed. For normally distributed data a one-way analysis of variance (ANOVA) followed by Holm-Sidak *post hoc* test for multiple comparisons was performed. Unpaired students t-test or Mann-Whitney test were used to compare the differences between two groups. Two-way ANOVA was used to compare two independent variables between two or more normally distributed groups. The specific tests that were used for each data set are reported in the relevant figure legends. A *p*-value of ≤0.05 was considered significant.

## Results

### Absence of Chronotropic, Dromotropic or Hemodynamic Effects of CB13 in the Non-Paced Rat Heart

**FIGURE 2:**
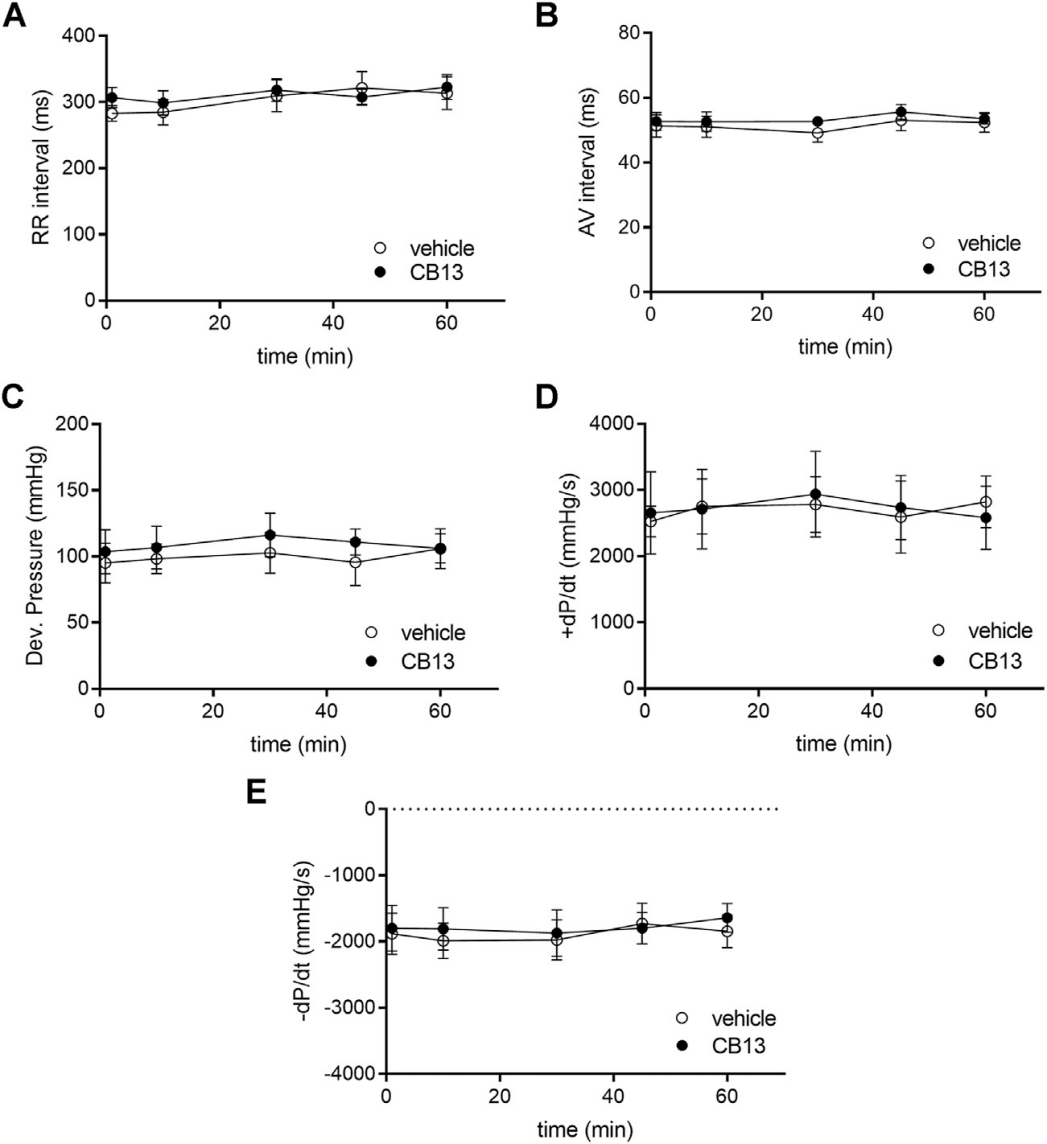
Absence of chronotropic, dromotropic or hemodynamic effects of CB13 in the non-paced rat heart. Isolated rat heart preparations were exposed to either 1 μM of CB13 or vehicle alone, and **(A)** RR interval, **(B)** atrioventricular (AV) interval, **(C)** developed pressure **(D)** maximal rate of rise of LVP (+dP/dt), and **(E)** maximal rate of decay of LVP (−dP/dt) were assessed. All parameters demonstrated stability over time with no difference between the CB13 and vehicle preparations. (*n* = 5). Data presented as mean ± SEM.

The physiological effects of CB13 on the intact heart were not determined previously. Thus, we began by evaluating the effects of the compound in isolated, non-paced rat hearts; the effects of CB13 (1 μM, *n* = 5) were compared to vehicle treatment (*n* = 5) in regard to heart rate (RR interval), AV conduction delay (AV interval) and LV hemodynamic properties over a period of 1 h (Figure 2). RR interval and AV interval remained stable throughout the experimental period without any significant differences at *t* = 60 min compared to vehicle (323.0 ± 19.0 ms vs. 313.4 ± 24.8, and 53.50 ± 1.59 ms vs. 52.35 ± 2.98 ms, respectively). Likewise, there was no change in LV developed pressure (106.0 ± 16.0 vs. 105.9 ± 15.1 mm Hg vehicle). Furthermore, positive and negative maximal slopes of the LVP contraction (+dP/dt, 2,581 ± 478 vs. 2,822 ± 392 mm Hg/s) or relaxation (−dP/dt, −1,654 ± 219 vs. −1,846 ± 248 mm Hg/s) were also maintained between groups. Overall, these findings demonstrate both stability in technical terms and lack of notable effects of CB13 on intact rat heart physiology.

### CB13-Treatment Inhibited Tachypacing-Induced Atrial Electrical Remodeling

**FIGURE 3:**
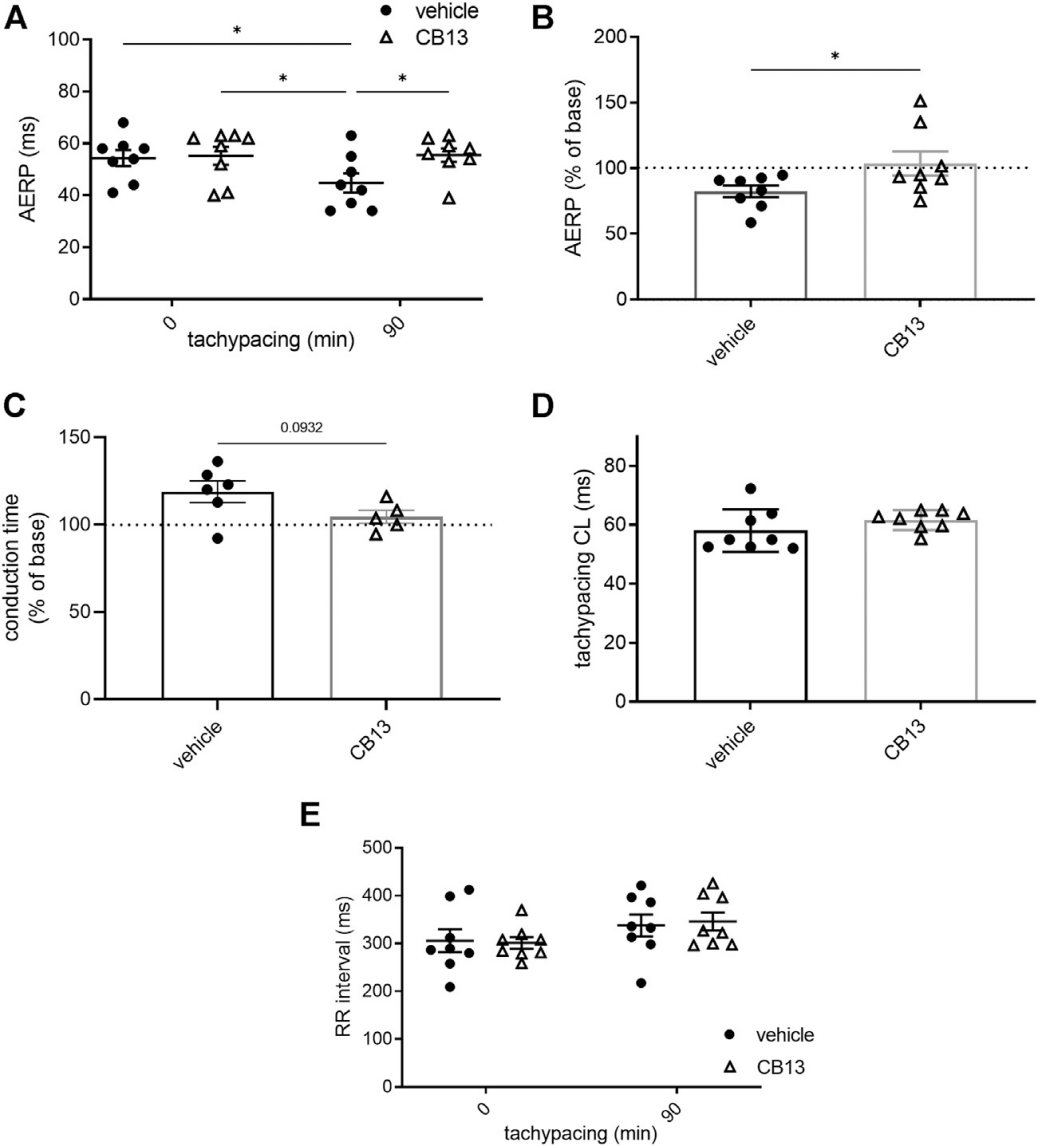
CB13-treatment inhibited tachypacing-induced atrial electrical remodeling. **(A)** AERP before and after 90 min of atrial tachypacing; **(B)** Scatter plot demonstrating the effect of atrial tachypacing on the AERP represented as % of baseline value in each experiment. CB13 treatment prevented reduction in AERP following tachypacing. **(C)** Conduction time analysis demonstrated a trend of lengthening in vehicle treated tachypaced hearts relative to the CB13 treated hearts. **(D)** Average pacing CL over the 90 min of atrial tachypacing. The CL was adjusted continuously and maintained 5 ms above the minimal CL to maintain 1:1 atrial capture. Note no difference between the CB13 group and vehicle group in this regard. **(E)** Spontaneous RR interval measured before and after 90 min of atrial tachypacing in both groups. Note a mild tendency of RR interval prolongation which reached significance in the CB13 group only. *n* = 5–8 for each group. Data are presented as mean ± SEM. *p ≤ 0.05. CL, cycle length; AERP, atrial effective refractory period. Based on the normality test statistical significance was determined using two-way ANOVA with Newman-Keuls test for multiple comparisons **(A,E)**, Mann-Whitney test **(B)**.

As previously described (Etzion et al., 2008), *ex vivo* atrial tachypacing reduced AERP in vehicle-treated hearts compared to baseline (45 ± 4 vs. 54 ± 3 ms; *p* < 0.05; Figure 3A). In contrast, the AERP of CB13-treated hearts remained unchanged after atrial tachypacing compared to baseline (56 ± 3 vs. 55 ± 3 ms; ns; Figure 3A). Therefore, during tachypacing, CB13 treatment preserved AERP, which contrasts with the AERP shortening observed in vehicle-treated hearts (103.6 ± 9.2 vs. 82.3 ± 4.4% of baseline, respectively; *p* < 0.05) (Figure 3B). Importantly, prior to tachypacing, there was no difference in AERP with CB13 perfusion compared to vehicle (55 ± 3 vs. 55 ± 3 ms, ns; Figure 3A). Of note, conduction time analysis demonstrated a trend of lengthening in vehicle treated tachypaced hearts relative to the CB13 treated hearts. However, this trend did not reach significance (*p* = 0.09, Figure 3C). Since tachypacing cycle length (CL) was adjusted during the pacing protocol (*see Methods* for details), it was important to verify that the CL did not differ between groups. Indeed, average atrial tachypacing CL was not different between the CB13 and vehicle groups (Figure 3D). Comparison of the spontaneous RR interval before and after 1.5 h of atrial tachypacing indicated a mild, statistically significant tendency of prolongation which did not differ significantly between the two treatments (Figure 3E). AF induction by burst pacing episodes failed to induce arrhythmia in both treatment groups both before and after the tachypacing (except in sporadic cases). Thus, the obtained results were inconclusive and are not shown.

### Biochemical Effects of CB13 in the Tachypaced Atria

**FIGURE 4:**
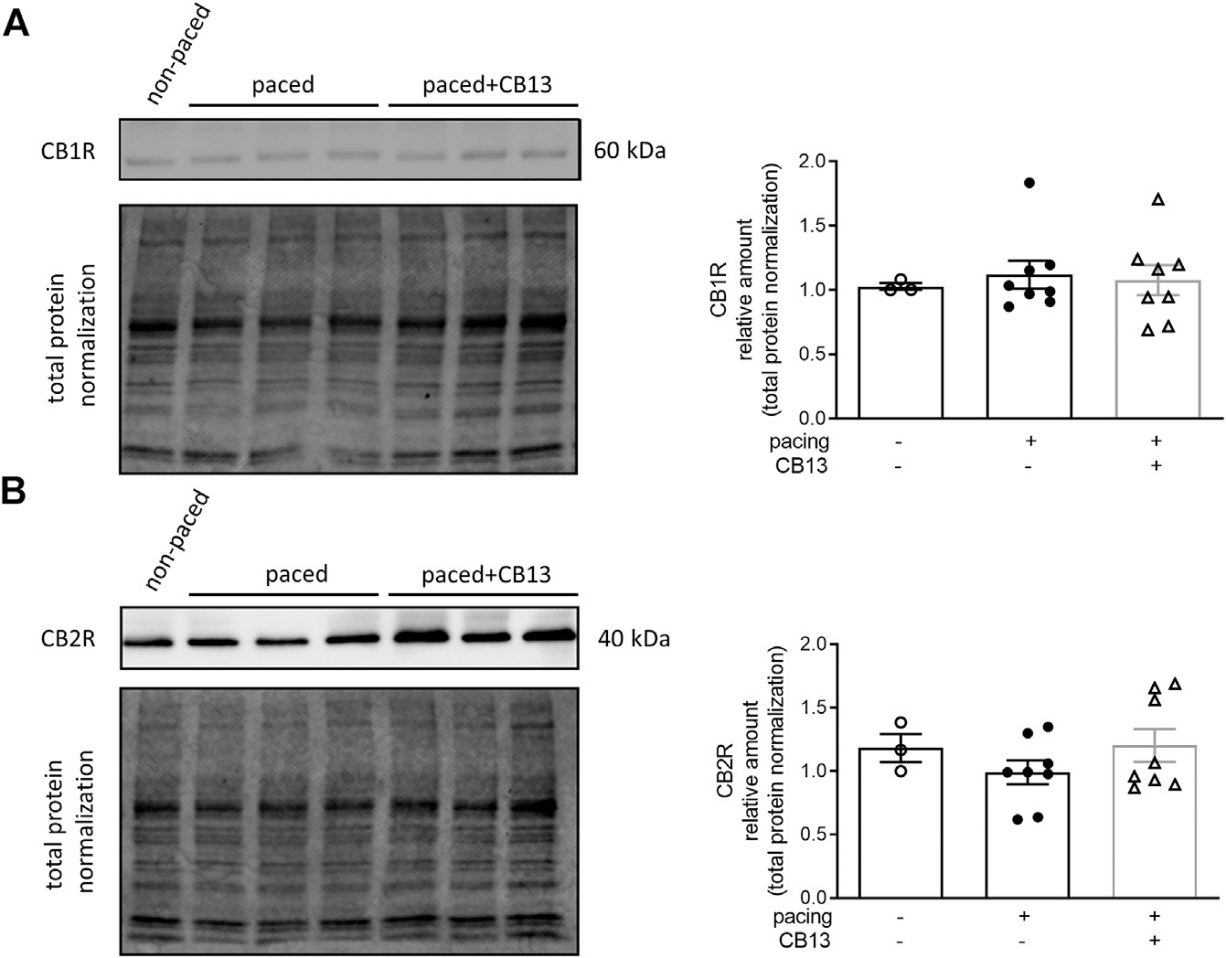
Cannabinoid receptor protein levels remain unaltered in atrial tissue. **(A)** CB1R and **(B)** CB2R protein levels within atrial tissue exhibit no significant changes between groups. *n* = 3–8. Data presented as mean ± SEM. Data was tested for normality using Shapiro-Wilk normality test. Statistical significance was determined for non-normal data by Kruskal-Wallis with Dunn’s post-hoc for multiple comparisons **(A,B)**.

**FIGURE 5:**
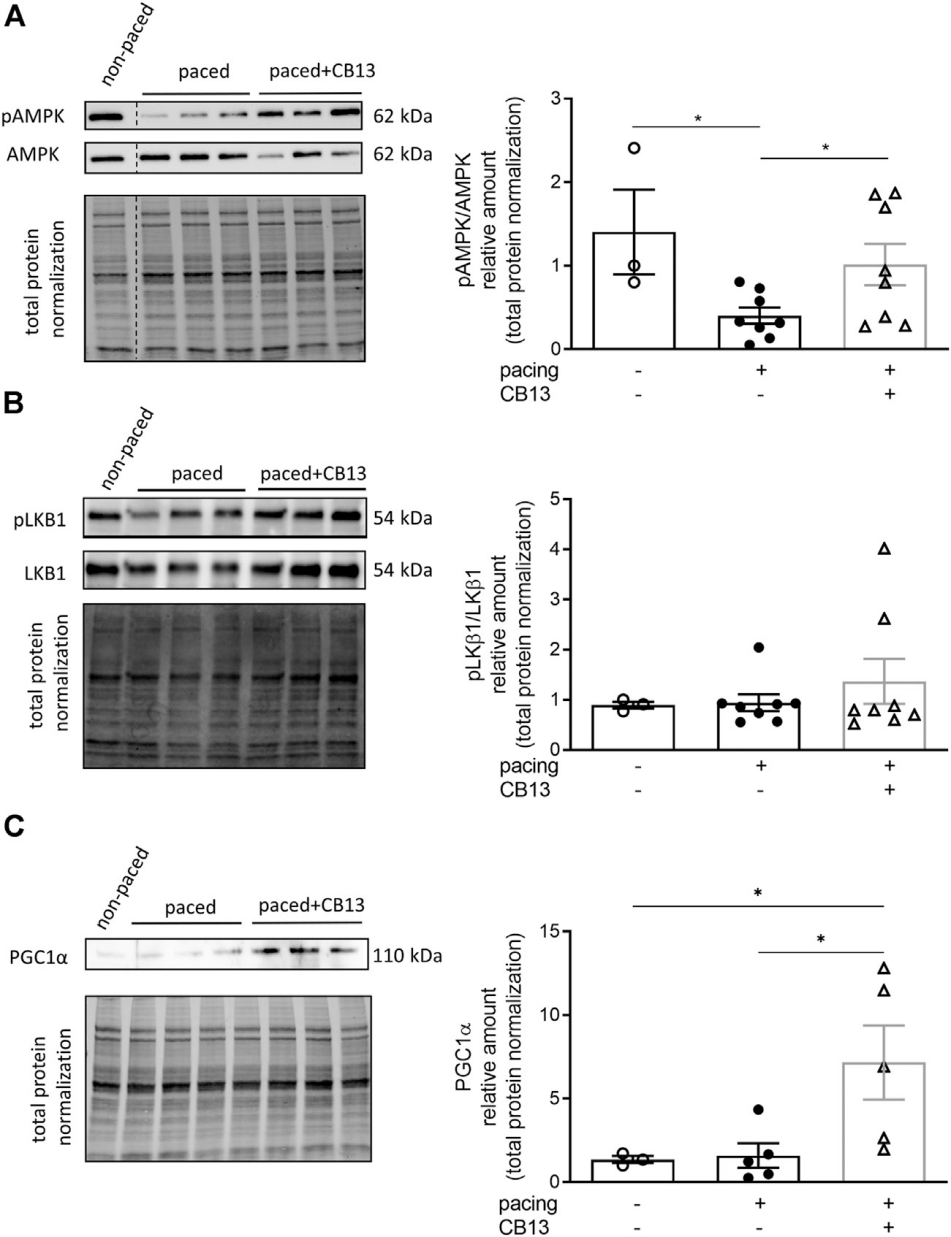
Tachypacing induced reduction of phosphorylated AMPK, while CB13 treatment abrogated tachypacing effects and increased PGC1α signaling. **(A)** Tachypacing for 1.5 h significantly reduced pAMPK compared to non-paced atrial tissue, while CB13 perfusion during tachypacing rescued pAMPK. **(B)** pLKB1 levels did not change between groups. **(C)** Protein expression levels of PGC1α were unaltered in both non-paced and tachypaced atrium whereas CB13 perfusion during tachypacing significantly upregulated PGC1α. *p ≤ 0.05. *n* = 3–8. Data presented as mean ± SEM. Data was tested for normality using Shapiro-Wilk normality test. Accordingly, statistical analysis was performed by one-way ANOVA with Holm-Sidak post-hoc for multiple comparisons **(A, C)** and by Kruskal-Wallis with Dunn’s post-hoc for multiple comparisons **(B)**.

**FIGURE 6:**
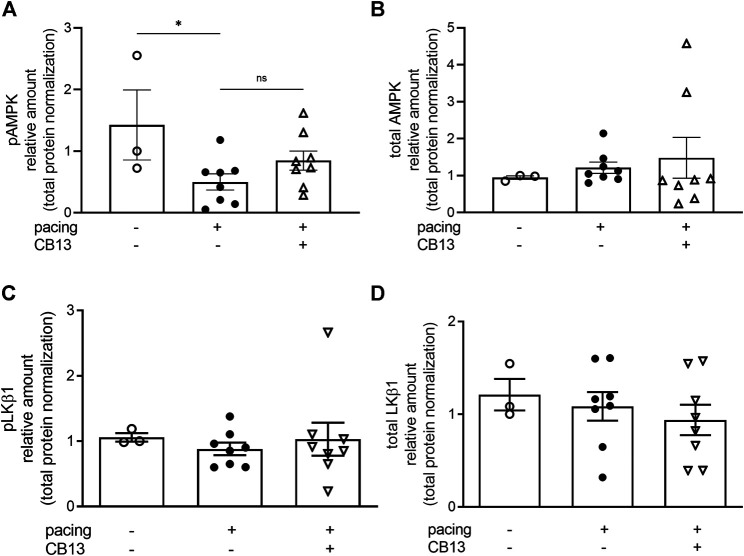
Total AMPK and total LKB1 were not altered in atrial tissue. **(A)** Tachypacing for 1.5 h significantly reduced pAMPK compared to non-paced atrial tissue **(B)** but did not alter total AMPK. **(C)** Phospho-LKB1 and **(D)** total LKB1 protein expression did not change between groups. See Figures 5A,B for representative blots. *p ≤ 0.05. ns; not significant. *n* = 3–8. Data presented as mean ± SEM. Data was tested for normality using Shapiro-Wilk normality test. Accordingly, statistical significance was determined by one-way ANOVA with Holm-Sidak post-hoc for multiple comparisons **(A)**.

In order to further reveal the effects of CB13 in the context of atrial tachypacing, biochemical analyses of relevant signalling effectors were performed in LA tissue homogenates. Protein expression levels of CB1R or CB2R did not differ between non-paced control tissues and tachypaced preparations (Figure 4), nor between CB13 and vehicle treatments during tachypacing (Figure 4). In contrast, tachypacing significantly reduced phosphorylation of AMPKα at Thr172, and this effect was partially attenuated by CB13 perfusion during tachypacing (Figure 5A). Neither tachypacing nor CB13 affected LKB1 expression or phosphorylation (Figure 5B), which indicates that the aforementioned changes in AMPK signaling are not induced through LKB1 modulation (*see Discussion*). PGC1α is phosphorylated by AMPK and acts as a fundamental co-activator for transcription of genes that regulate mitochondrial function. Expression levels of PGC1α were comparable in non-paced and tachypaced atrium (Figure 5C). However, tachypacing in the presence of CB13 upregulated PGC1α compared to both non-paced and tachypaced atrium (Figure 5C). Of note, there were no changes in total AMPK or total LKB1 levels in our experimental settings (Figure 6).

**FIGURE 7:**
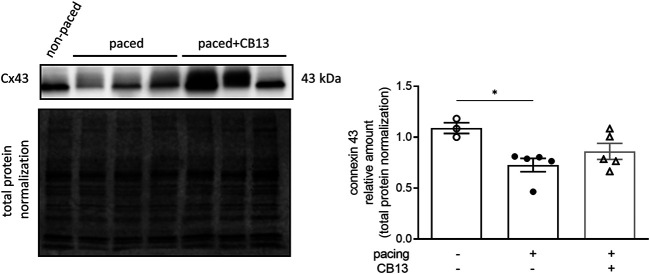
Tachypacing-induced reduction of connexin 43 is diminished by CB13. (A) Cx43 is downregulated in tachypaced atrium compared to non-paced atrium. This effect of tachypacing demonstrated a lower tendency in the presence of CB13. *p ≤ 0.05. *n* = 3–5. Data presented as mean ± SEM. Data was tested for normality using Shapiro-Wilk normality test. Accordingly, statistical analysis was performed by Kruskal-Wallis with Dunn’s post-hoc for multiple comparisons.

Connexin 43 (Cx43) is a major gap junction protein in the heart which was recently linked to AMPK signaling in the context of AF (Qiu et al., 2016b). Cx43 was downregulated in tachypaced atria compared to non-paced controls (Figure 7). Although, there appeared to be a trend of recovery of this effect in the presence of CB13, this effect did not reach statistical significance.

## Discussion

The present study extends extant evidence indicating possible beneficial effects of ECS activation in the stressed myocardium (Wagner et al., 2001; Lu et al., 2014; Lu et al., 2020). Overall, this study demonstrated three salient findings regarding the cardiac effects of the peripherally restricted, dual CBR agonist CB13 in rats: 1) lack of CB13-dependent chronotropic, dromotropic or hemodynamic effects in the non-paced *ex-vivo* preparation; 2) an ability of CB13 to antagonize the AERP remodelling induced by acute atrial tachypacing; 3) AMPK activation may contribute to the ability of CB13 to protect against tachypacing-induced atrial remodelling. These topics are further discussed in detail below.

Our findings address the paucity of knowledge regarding the direct effects of CBR agonists on the intrinsic properties of the intact heart (Weresa et al., 2019), and support the notion that combined activation of CB1R and CB2R probably does not markedly alter the basic electrophysiology, calcium handling and electromechanical coupling of the normal rat myocardium. Indeed, our data indicate that CB13 treatment does not alter chronotropy, dromotropy, or hemodynamics in the spontaneously beating rat heart. Activation of solely CB1Rs reportedly induces negative inotropic effects in isolated human atrial muscle (Bonz et al., 2003). Likewise, the CB1R agonist, HU-210, induced negative inotropic effects in the LV of the isolated rat heart (Krylatov et al., 2005), although a recent study also using HU-210 did not report such effect (Gorbunov et al., 2016). Interestingly, in the presence of the CB1R antagonist AM251, anandamide exerted a CB2R-mediated positive inotropic effect in rat atria (Sterin-Borda et al., 2005). Thus, it is conceivable that the absence of CB13 effects on intrinsic cardiac properties results from opposing effects of CB1R activation and CB2R activation which manifest as a net zero effect. This is consistent with previous reports of opposing CB1 *vs.* CB2 effects. In fact, CB2R activation has been linked to cardioprotective effects including anti-arrhythmic effects (Krylatov et al., 2001), whereas CB1R activation leads to a myriad of cardiodeleterious effects (Mukhopadhyay et al., 2007; Dol-Gleizes et al., 2009; Sugamura et al., 2009; Mukhopadhyay et al., 2010; Rajesh et al., 2010; Tiyerili et al., 2010; Rajesh et al., 2012; Molica et al., 2013). Thus, Lu et al proposed that, in the context of attenuating cardiac myocyte hypertrophy, activation of CB2Rs is necessary to negate any adverse effects produced by CB1Rs. Further pharmacological studies will be required to systematically explore this possibility in terms of chronotropy, dromotropy, or hemodynamics in the spontaneously beating rat heart.

Atrial tachypacing is an important experimental approach to mimic AF-related remodeling. In large mammals, sustained tachypacing modifies atrial properties so that AF susceptibility and sustainability gradually increase over time (Wijffels et al., 1995; Nattel et al., 2020). Studies have indicated that the rapid atrial activity and consequent cellular calcium overload within atrial cardiomyocytes lead to secondary changes which converge to increase AF substrate. Shortening of AERP is a hallmark of AF-related remodeling and an important factor that promotes multiple circuit reentry in the atria (Wakili et al., 2011; Nattel et al., 2020). In rodents the long-term effects of atrial tachypacing are less clear. However, short-term tachypacing consistently leads to AERP shortening (Etzion et al., 2008). In addition, we recently demonstrated that atrial tachypacing of freely moving rats increases AF substrate and promotes molecular changes that resemble those reported in large mammalian models (Mulla et al., 2019). Moreover, tachypacing of atrial cells *in vitro* leads to molecular changes that recapitulate those observed in the atrial tissue of AF patients (Wiersma et al., 2017; Zhang et al., 2019). Our current findings indicate that CB13 inhibits the AERP shortening induced by acute atrial tachypacing in the *ex vivo* rat heart. This finding supports the notion that activation of CBRs may be a new therapeutic target in the context of AF-related remodelling. However, further studies are needed to delineate the long-term effects of this new therapeutic strategy *in vivo*. In addition, since AF susceptibility of the normal rat heart is very low, it is challenging to induce AF even following atrial tachypacing and AERP shortening in the current experimental setup. Therefore, the effect of CB13 on AF induction will rely on future preparations that are more prone to AF induction (e.g., the atria of rats with heart failure post myocardial infarction) (Klapper-Goldstein et al., 2020).

The biochemical findings of our study suggest that AMPK activation is involved in the protective effect of CB13 against tachypacing-induced atrial remodeling. AMPK is an essential signaling molecule that contributes to the maintenance of intracellular ATP levels (Dolinsky and Dyck, 2006). To this end, AMPK controls energy homeostasis and senses changes in AMP:ATP to in turn control ATP-consumption and catabolic pathways(Dolinsky and Dyck, 2006). Reduced AMPK activation in atrial cardiomyocytes appears to be deleterious in regards to regulating metabolic and oxidative stress (Ozcan et al., 2015). Importantly, multiple risk factors for AF such as heart failure, myocardial ischemia, and hypertrophy are associated with metabolic stress and cellular dysfunction (Harada et al., 2012). By virtue of ameliorating these metabolic abnormalities, AMPK activation may thus be implicated in improving arrhythmias (Harada et al., 2012). In fact, Ozcan *et al* demonstrated that liver kinase-B1 (LKB1; an AMPK kinase that is linked to activation of AMPK) knockout mice exhibit impaired contraction and decreased AMPK activity (Ozcan et al., 2015). This LKB1 knockout model replicates human disease progression as it develops spontaneous AF that develops into persistent AF (Ozcan et al., 2015).

Our data indicate that acute atrial tachypacing *ex vivo* leads to downregulation of phosphorylated AMPK, similar to that found in humans experiencing persistent AF (Harada et al., 2015). In this context, CB13 abrogated AMPK downregulation and increased phosphorylation at its activation site, presumably improving metabolic and cellular function. Although we did not demonstrate a direct link between the electrophysiological effects and metabolic findings, we speculate that improved metabolic conditions of atrial cells in the presence of CB13 lead to less oxidative stress-dependent AERP shortening (Korantzopoulos et al., 2007; Yoshizawa et al., 2018). However, this hypothesis will have to be addressed more directly in future studies. Furthermore, AERP reduction may also be caused by other factors; for example Ozgen *et al* demonstrated involvement of Ca^2+^-activated K^+^ currents in action potential duration and atrial remodeling (Ozgen et al., 2007). Additionally, AMPK activation by CB13 is likely not mediated through LKB1 activation (Figure 5B) and is therefore likely achieved through changes in cellular Ca^2+^ levels, particularly since Ca^2+^/calmodulin-dependent protein kinase kinases (CAMKKs) and reactive oxygen species (ROS), which promote AF, have been implicated to alter AMPK signalling (Lin et al., 2004; Kim et al., 2016). In fact, CAMKKα and CAMKKβ phosphorylate AMPK at its activation site (Woods et al., 2003; Hawley et al., 2005; Hurley et al., 2005), and CBR ligands can signal through CAMKKs (Vara et al., 2011). An important future direction will be to utilize a CAMKK inhibitor to validate this hypothesis.

Information regarding the mediator between AMPK and arrhythmia development, tissue remodeling, or modification of AF substrate remains limited(Harada et al., 2012). However, there does appear to be a relationship between cardiac EP and metabolic changes that involve a key role of AMPK and AF substrate (Qiu et al., 2016a; Qiu et al., 2016b). In the current experimental setup, we identified PGC-1α as a candidate mediator for CB13-dependent effects (Figure 5C). PGC-1α regulates mitochondrial function vis-à-vis its function as a key transcriptional co-activator that modulates mitochondrial biogenesis, ATP synthesis, and reactive oxygen species (ROS) defense mechanisms (Ventura-Clapier et al., 2008; St-Pierre et al., 2006). Activation of AMPK results in a downstream cascade that increases PGC-1α expression (Jager et al., 2007). Notably, PGC-1α overexpression rescues cardiac mitochondrial function (Zhou et al., 2013). Tachypacing had no effect on PGC-1α compared to non-pacing controls (Figure 5C). However, CB13 treatment caused a significant upregulation of PGC-1α. We previously speculated that CB13 activates AMPK via CB2R whereas CB1 receptors invoke other signaling pathways (Lu et al., 2020). In addition, Zheng et al. demonstrated that JWH-133, a CB2R-selective agonist, adequately activated AMPK to stimulate PGC-1α, without CB1R interaction (Zheng et al., 2013). Here we elucidated that CB13 treatment during tachypacing induces a CBR/AMPK (possibly CB2R, specifically) signaling cascade, resulting in PGC-1α activation. While we have previously shown that CB13 has altered mitochondrial bioenergetics and mitochondrial membrane potential in hypertrophied ventricular cardiomyocytes, an important next step is to determine the effects of CB13 on mitochondrial dysfunction in atrial cardiomyocytes, and the role of the AMPK signaling cascade therein.

Lastly, our data indicate that tachypacing reduces the atrial expression of Cx43, and this reduction was attenuated by with CB13 treatment. Cx43 is a major gap junction in the connexin family and is abundantly found within the atrial and ventricular myocardium (Severs et al., 2008). Cx43 has been investigated in several AF-related remodelling studies; for example, in dog pacing models Cx43 reduction has been demonstrated after tachypacing for 3 days, with reduction being preserved for up to 21 days (Akar et al., 2007). In rabbit atrial tachypacing experiments Cx43 reduction has been shown to promote atrial fibrillation development (Yan et al., 2013). Additionally, Ozcan *et al* demonstrated that AMPK activation may inhibit atrial fibrillation in LKB1 knockout mice, and similarly found LKB1 knockout mice had a reduction in Cx43 that was rescued by AMPK activation (Ozcan et al., 2020). In contrast, Alesutan *et al* demonstrated inhibition of Cx43 by AMPK (Alesutan et al., 2015); thus Cx43 and its interaction with AMPK in the context of electrical remodeling remains inconclusive. Furthermore, downregulation of Cx43 would lead to reduced EP coupling between atrial myocytes, which in turn would slow conduction velocity and increase AF susceptibility in atrial tissue (Luo et al., 2018). While we did not demonstrate a change in conduction velocity between groups there was a trend towards conduction lengthening in the vehicle treatment group. It is possible that the short-term tachypacing in our model did not allow for more prominent effects to be observed. Of note, oxidative stress associated with rapid atrial activity has been shown to cause Cx43 alterations (Boengler and Schulz, 2017; Nattel et al., 2020); and this may be modulated by AMPK activation (Qiu et al., 2016b). Further studies to examine the interplay between CB13, AMPK, and Cx43 are clearly warranted.

In conclusion, our data demonstrating the ability of CB13 treatment to prevent tachypacing-induced AERP shortening and Cx43 reduction, as well as to activate known cardioprotective signaling mediators in terms of metabolic dysfunction (i.e., AMPK and PGC-1 α), are collectively in favor of a putative beneficial effect of this drug as a new upstream modality that might be utilized to prevent atrial electrical remodeling. Considering the possible beneficial effects of such treatment in the context of pathological hypertrophy (Lu et al., 2014; Lu et al., 2020), which shares many of the risk factors for AF, this therapeutic strategy is even more attractive for further investigation.

### Limitations

Electrophysiologic properties of the atria differ between that of rodents and humans, and thus represent a limitation of our study. Dependent on species, the atria have altered electrophysiological properties, including action potential duration and action potential repolarization (Clauss et al., 2019). Nevertheless, the relevance of rodent models to AF studies are becoming increasingly recognized (Nattel et al., 2005; Nishida et al., 2010; Klapper-Goldstein et al., 2020). In addition, there are multiple studies indicating that tachypacing induces oxidative stress and affects the molecular biology of rodent cardiomyocytes in a highly clinically relevant manner. (Gao et al., 2011; Mulla et al., 2019; Wiersma et al., 2019; Zhang et al., 2019). Furthermore, while we suspect that AMPK activation is linked to ameliorating metabolic abnormalities and improving atrial remodeling through mechanisms including altering AERP, the exact link between AERP and AMPK activation remains to be determined. Assessment of activation status (i.e. phosphorylated levels) of AMPK and presumably LKB1 is reasonable within 1.5 h, and the ability of CB13 to increase PGC1 is likewise plausible as a downstream effector of AMPK signaling. However, detection of changes in CB1R and CB2R expression levels might require more than 1.5 h of experimental intervention. Further experiments will be crucial to elucidate the underlying mechanisms, and to delineate relevant ionic mechanisms. Of note, we recently introduced a methodology enabling comprehensive EP measurements and AF susceptibility testing in ambulatory rats over time (Murninkas et al., 2021). Thus, based on the current findings it will be interesting to explore the effects of CB13 on longer periods of tachypacing in unanesthetized rats in the near future.

## Data Availability

The original contributions presented in the study are included in the article. Further inquiries can be directed to the corresponding authors.

## References

[B1] Akar F. G. Nass R. D. Hahn S. Cingolani E. Shah M. Hesketh G. G. DiSilvestre D. Tunin R. S. Kass D. A. Tomaselli G. F. (2007). Dynamic changes in conduction velocity and gap junction properties during development of pacing-induced heart failure. Am. J. Physiology-Heart Circulatory Physiol. 293 (2), H1223–H1230. 10.1152/ajpheart.00079.2007 17434978

[B2] Alesutan I. Voelkl J. Stöckigt F. Mia S. Feger M. Primessnig U. Sopjani M. Munoz C. Borst O. Gawaz M. Pieske B. Metzler B. Heinzel F. Schrickel J. W. Lang F. (2015). AMP-activated protein kinase α1 regulates cardiac gap junction protein connexin 43 and electrical remodeling following pressure overload. Cell Physiol Biochem 35 (1), 406–418. 10.1159/000369706 25591781

[B3] Anter E. Di Biase L. Contreras-Valdes F. M. Gianni C. Mohanty S. Tschabrunn C. M. (2017). Atrial substrate and triggers of paroxysmal atrial fibrillation in patients with obstructive sleep apnea. Circ. Arrhythm Electrophysiol. 10 (11), e005407. 10.1161/circep.117.005407 29133380 PMC5726812

[B4] Boengler K. Schulz R. (2017). Connexin 43 and mitochondria in cardiovascular health and disease. Adv. Exp. Med. Biol. 982, 227–246. 10.1007/978-3-319-55330-6_12 28551790

[B5] Bonz A. Laser M. Küllmer S. Kniesch S. Babin-Ebell J. Popp V. Ertl G. Wagner J. A. (2003). Cannabinoids acting on CB1 receptors decrease contractile performance in human atrial muscle. J. Cardiovasc. Pharmacol. 41 (4), 657–664. 10.1097/00005344-200304000-00020 12658069

[B6] Bouchard J.-F. Lépicier P. Lamontagne D. (2003). Contribution of endocannabinoids in the endothelial protection afforded by ischemic preconditioning in the isolated rat heart. Life Sci. 72 (16), 1859–1870. 10.1016/s0024-3205(02)02474-8 12586223

[B7] Clauss S. Bleyer C. Schüttler D. Tomsits P. Renner S. Klymiuk N. Wakili R. Massberg S. Wolf E. Kääb S. (2019). Animal models of arrhythmia: classic electrophysiology to genetically modified large animals. Nat. Rev. Cardiol. 16, 457–475. 10.1038/s41569-019-0179-0 30894679

[B8] Dobrev D. Nattel S. (2010). New antiarrhythmic drugs for treatment of atrial fibrillation. The Lancet 375 (9721), 1212–1223. 10.1016/s0140-6736(10)60096-7 20334907

[B9] Dol-Gleizes F. Paumelle R. Visentin V. Marés A.-M. Desitter P. Hennuyer N. Gilde A. Staels B. Schaeffer P. Bono F. (2009). Rimonabant, a selective cannabinoid CB1 receptor antagonist, inhibits atherosclerosis in LDL receptor-deficient mice. Atvb 29 (1), 12–18. 10.1161/atvbaha.108.168757 18845788

[B10] Dolinsky V. W. Dyck J. R. B. (2006). Role of AMP-activated protein kinase in healthy and diseased hearts. Am. J. Physiology-Heart Circulatory Physiol. 291 (6), H2557–H2569. 10.1152/ajpheart.00329.2006 16844922

[B11] Ehrlich J. R. Nattel S. Hohnloser S. H. (2002). Atrial fibrillation and congestive heart failure: specific considerations at the intersection of two common and important cardiac disease sets. J. Cardiovasc. Electrophysiol. 13 (4), 399–405. 10.1046/j.1540-8167.2002.00399.x 12033360

[B12] Etzion Y. Mor M. Shalev A. Dror S. Etzion O. Dagan A. Beharier O. Moran A. Katz A. (2008). New insights into the atrial electrophysiology of rodents using a novel modality: the miniature-bipolar hook electrode. Am. J. Physiology-Heart Circulatory Physiol. 295 (4), H1460–H1469. 10.1152/ajpheart.00414.2008 18660446

[B13] Galiegue S. Mary S. Marchand J. Dussossoy D. Carriere D. Carayon P. Bouaboula M. Shire D. Fur G. Casellas P. (1995). Expression of central and peripheral cannabinoid receptors in human immune tissues and leukocyte subpopulations. Eur. J. Biochem. 232 (1), 54–61. 10.1111/j.1432-1033.1995.tb20780.x 7556170

[B14] Gao S. Yuan K. Shah A. Kim J. S. Park W. H. Kim S. H. (2011). Suppression of high pacing-induced ANP secretion by antioxidants in isolated rat atria. Peptides 32 (12), 2467–2473. 10.1016/j.peptides.2011.10.022 22063193

[B15] Gebremedhin D. Lange A. R. Campbell W. B. Hillard C. J. Harder D. R. (1999). Cannabinoid CB1 receptor of cat cerebral arterial muscle functions to inhibit L-type Ca2+ channel current. Am. J. Physiology-Heart Circulatory Physiol. 276 (6 Pt 2), H2085–H2093. 10.1152/ajpheart.1999.276.6.h2085 10362691

[B16] Gorbunov A. S. Maslov L. N. Tsibulnikov S. Y. Khaliulin I. G. Tsepokina A. V. Khutornaya M. V. Kutikhin A. G. (2016). CB-receptor agonist HU-210 mimics the postconditioning phenomenon of isolated heart. Bull. Exp. Biol. Med. 162 (1), 27–29. 10.1007/s10517-016-3536-6 27878734

[B17] Harada M. Nattel S. N. Nattel S. (2012). AMP-activated protein kinase. Circ. Arrhythm Electrophysiol. 5 (4), 860–867. 10.1161/circep.112.972265 22895602

[B18] Harada M. Tadevosyan A. Qi X. Xiao J. Liu T. Voigt N. Karck M. Kamler M. Kodama I. Murohara T. Dobrev D. Nattel S. (2015). Atrial fibrillation activates AMP-dependent protein kinase and its regulation of cellular calcium handling. J. Am. Coll. Cardiol. 66 (1), 47–58. 10.1016/j.jacc.2015.04.056 26139058

[B19] Hawley S. A. Pan D. A. Mustard K. J. Ross L. Bain J. Edelman A. M. Frenguelli B. G. Hardie D. G. (2005). Calmodulin-dependent protein kinase kinase-β is an alternative upstream kinase for AMP-activated protein kinase. Cel Metab. 2 (1), 9–19. 10.1016/j.cmet.2005.05.009 16054095

[B20] Hayashi K. Tada H. Yamagishi M. (2017). The genetics of atrial fibrillation. Curr. Opin. Cardiol. 32 (1), 10–16. 10.1097/hco.0000000000000356 27861186

[B21] Heijman J. Algalarrondo V. Voigt N. Melka J. Wehrens X. H. T. Dobrev D. Nattel S. (2016). The value of basic research insights into atrial fibrillation mechanisms as a guide to therapeutic innovation: a critical analysis. Cardiovasc. Res. 109 (4), 467–479. 10.1093/cvr/cvv275 26705366 PMC4777910

[B22] Hurley R. L. Anderson K. A. Franzone J. M. Kemp B. E. Means A. R. Witters L. A. (2005). The Ca2+/calmodulin-dependent protein kinase kinases are AMP-activated protein kinase kinases. J. Biol. Chem. 280 (32), 29060–29066. 10.1074/jbc.m503824200 15980064

[B23] Jager S. Handschin C. St.-Pierre J. Spiegelman B. M. (2007). AMP-activated protein kinase (AMPK) action in skeletal muscle via direct phosphorylation of PGC-1. Proc. Natl. Acad. Sci. 104 (29), 12017–12022. 10.1073/pnas.0705070104 17609368 PMC1924552

[B24] Kim J. Yang G. Kim Y. Kim J. Ha J. (2016). AMPK activators: mechanisms of action and physiological activities. Exp. Mol. Med. 48, e224. 10.1038/emm.2016.16 27034026 PMC4855276

[B25] Klapper-Goldstein H. Murninkas M. Gillis R. Mulla W. Levanon E. Elyagon S. (2020). An implantable system for long-term assessment of atrial fibrillation substrate in unanesthetized rats exposed to underlying pathological conditions. Sci. Rep. 10 (1), 553. 10.1038/s41598-020-57528-3 31953473 PMC6969190

[B26] Korantzopoulos P. Kolettis T. M. Galaris D. Goudevenos J. A. (2007). The role of oxidative stress in the pathogenesis and perpetuation of atrial fibrillation. Int. J. Cardiol. 115 (2), 135–143. 10.1016/j.ijcard.2006.04.026 16764958

[B28] Krylatov A. V. Maslov L. N. Lasukova O. V. Pertwee R. G. (2005). Cannabinoid receptor antagonists SR141716 and SR144528 exhibit properties of partial agonists in experiments on isolated perfused rat heart. Bull. Exp. Biol. Med. 139 (5), 558–561. 10.1007/s10517-005-0344-9 16224548

[B27] Krylatov A. V. Ugdyzhekova D. S. Bernatskaya N. A. Maslov L. N. Mekhoulam R. Pertwee R. G. Stephano G. B. (2001). Activation of type II cannabinoid receptors improves myocardial tolerance to arrhythmogenic effects of coronary occlusion and reperfusion. Bull. Exp. Biol. Med. 131 (6), 523–525. 10.1023/a:1012381914518 11586395

[B29] Le Boisselier R. Alexandre J. Lelong-Boulouard V. Debruyne D. (2017). Focus on cannabinoids and synthetic cannabinoids. Clin. Pharmacol. Ther. 101 (2), 220–229. 10.1002/cpt.563 27861784

[B30] Lee T. W. Lee T. I. Lin Y. K. Chen Y. C. Kao Y. H. Chen Y. J. (2020). Effect of antidiabetic drugs on the risk of atrial fibrillation: mechanistic insights from clinical evidence and translational studies. Cell Mol Life Sci. 78(3), 923-934. 10.1007/s00018-020-03648-y 32965513 PMC11072414

[B31] Li N. Brundel B. J. J. M. (2020). Inflammasomes and proteostasis novel molecular mechanisms associated with atrial fibrillation. Circ. Res. 127 (1), 73–90. 10.1161/circresaha.119.316364 32717176 PMC7388703

[B32] Lin C.-C. Lin J.-L. Lin C.-S. Tsai M.-C. Su M.-J. Lai L.-P. Huang S. K. S. (2004). Activation of the calcineurin-nuclear factor of activated T-cell signal transduction pathway in atrial fibrillation. Chest 126 (6), 1926–1932. 10.1016/s0012-3692(15)31443-4 15596694

[B33] Lippi G. Sanchis-Gomar F. Cervellin G. (2020). Global epidemiology of atrial fibrillation: an increasing epidemic and public health challenge. Int. J. Stroke, 16(2), 217-221. 10.1177/1747493019897870 31955707

[B34] Liu J. Gao B. Mirshahi F. Sanyal A. J. Khanolkar A. D. Makriyannis A. Kunos G. (2000). Functional CB1 cannabinoid receptors in human vascular endothelial cells. Biochem. J. 346, 835–840. 10.1042/0264-6021:3460835 10698714 PMC1220920

[B35] Lu Y. Akinwumi B. C. Shao Z. Anderson H. D. (2014). Ligand activation of cannabinoid receptors attenuates hypertrophy of neonatal rat cardiomyocytes. J. Cardiovasc. Pharmacol. 64 (5), 420–430. 10.1097/fjc.0000000000000134 24979612

[B36] Lu Y. Lee D. I. Roy Chowdhury S. Lu P. Kamboj A. Anderson C. M. Fernyhough P. Anderson H. D. (2020). Activation of cannabinoid receptors attenuates endothelin-1-induced mitochondrial dysfunction in rat ventricular myocytes. J. Cardiovasc. Pharmacol. 75 (1), 54–63. 10.1097/fjc.0000000000000758 31815823 PMC6964873

[B37] Luo B. Yan Y. Zeng Z. Zhang Z. Liu H. Liu H. Li J. Huang W. Wu J. He Y. (2018). Connexin 43 reduces susceptibility to sympathetic atrial fibrillation. Int. J. Mol. Med. 42 (2), 1125–1133. 10.3892/ijmm.2018.3648 29717772

[B38] Matsuda L. A. Lolait S. J. Brownstein M. J. Young A. C. Bonner T. I. (1990). Structure of a cannabinoid receptor and functional expression of the cloned cDNA. Nature 346 (6284), 561–564. 10.1038/346561a0 2165569

[B39] Molica F. Burger F. Thomas A. Staub C. Tailleux A. Staels B. Pelli G. Zimmer A. Cravatt B. Matter C. M. Pacher P. Steffens S. (2013). Endogenous cannabinoid receptor CB1 activation promotes vascular smooth-muscle cell proliferation and neointima formation. J. Lipid Res. 54 (5), 1360–1368. 10.1194/jlr.m035147 23479425 PMC3622330

[B40] Montecucco F. Di Marzo V. (2012). At the heart of the matter: the endocannabinoid system in cardiovascular function and dysfunction. Trends Pharmacol. Sci. 33 (6), 331–340. 10.1016/j.tips.2012.03.002 22503477

[B41] Mor M. Shalev A. Dror S. Pikovsky O. Beharier O. Moran A. Katz A. Etzion Y. (2013). INO-8875, a highly selective A1 adenosine receptor agonist: evaluation of chronotropic, dromotropic, and hemodynamic effects in rats. J. Pharmacol. Exp. Ther. 344 (1), 59–67. 10.1124/jpet.112.200873 23055540

[B42] Moreno E. Cavic M. Krivokuca A. Casado V. Canela E. (2019). The endocannabinoid system as a target in cancer diseases: are we there yet?. Front. Pharmacol. 10, 339. 10.3389/fphar.2019.00339 31024307 PMC6459931

[B43] Mukhopadhyay P. Bátkai S. Rajesh M. Czifra N. Harvey-White J. Haskó G. Zsengeller Z. Gerard N. P. Liaudet L. Kunos G. Pacher P. (2007). Pharmacological inhibition of CB1Cannabinoid receptor protects against doxorubicin-induced cardiotoxicity. J. Am. Coll. Cardiol. 50 (6), 528–536. 10.1016/j.jacc.2007.03.057 17678736 PMC2239316

[B44] Mukhopadhyay P. Rajesh M. Bátkai S. Patel V. Kashiwaya Y. Liaudet L. Evgenov O. V. Mackie K. Haskó G. Pacher P. (2010). CB1 cannabinoid receptors promote oxidative stress and cell death in murine models of doxorubicin-induced cardiomyopathy and in human cardiomyocytes. Cardiovasc. Res. 85 (4), 773–784. 10.1093/cvr/cvp369 19942623 PMC2819835

[B45] Mulla W. Hajaj B. Elyagon S. Mor M. Gillis R. Murninkas M. (2019). Rapid atrial pacing promotes atrial fibrillation substrate in unanesthetized instrumented rats. Front. Physiol. 10, 1218. 10.3389/fphys.2019.01218 31616316 PMC6763969

[B46] Munro S. Thomas K. L. Abu-Shaar M. (1993). Molecular characterization of a peripheral receptor for cannabinoids. Nature 365 (6441), 61–65. 10.1038/365061a0 7689702

[B47] Murninkas M. Gillis R. Lee D. I. Elyagon S. Bhandarkar N. S. Levi O. Polak R. Klapper-Goldstein H. Mulla W. Etzion Y. (2021). A new implantable tool for repeated assessment of supraventricular electrophysiology and atrial fibrillation susceptibility in freely moving rats. Am. J. Physiology-Heart Circulatory Physiol. 320, H713. 10.1152/ajpheart.00676.2020 33337966

[B48] Nattel S. Dobrev D. (2017). Controversies about atrial fibrillation mechanisms. Circ. Res. 120 (9), 1396–1398. 10.1161/circresaha.116.310489 28450363

[B49] Nattel S. Harada M. (2014). Atrial remodeling and atrial fibrillation. J. Am. Coll. Cardiol. 63 (22), 2335–2345. 10.1016/j.jacc.2014.02.555 24613319

[B51] Nattel S. Heijman J. Zhou L. Dobrev D. (2020). Molecular basis of atrial fibrillation pathophysiology and therapy. Circ. Res. 127 (1), 51–72. 10.1161/circresaha.120.316363 32717172 PMC7398486

[B50] Nattel S. Shiroshita-Takeshita A. Brundel B. J. J. M. Rivard L. (2005). Mechanisms of atrial fibrillation: lessons from animal models. Prog. Cardiovasc. Dis. 48 (1), 9–28. 10.1016/j.pcad.2005.06.002 16194689

[B52] Nishida K. Michael G. Dobrev D. Nattel S. (2010). Animal models for atrial fibrillation: clinical insights and scientific opportunities. Europace 12 (2), 160–172. 10.1093/europace/eup328 19875395

[B53] Ozcan C. Battaglia E. Young R. Suzuki G. (2015). LKB1 knockout mouse develops spontaneous atrial fibrillation and provides mechanistic insights into human disease process. J. Am. Heart Assoc. 4 (3), e001733. 10.1161/jaha.114.001733 25773299 PMC4392447

[B54] Ozcan C. Dixit G. Li Z. (2020). Activation of AMP-activated protein kinases prevents atrial fibrillation. J. Cardiovasc. Transl Res. 10.1007/s12265-020-10069-6 32844365

[B55] Ozgen N. Dun W. Sosunov E. Anyukhovsky E. Hirose M. Duffy H. Boyden P. Rosen M. (2007). Early electrical remodeling in rabbit pulmonary vein results from trafficking of intracellular SK2 channels to membrane sites. Cardiovasc. Res. 75 (4), 758–769. 10.1016/j.cardiores.2007.05.008 17588552 PMC2034441

[B56] Pertwee R. G. Howlett A. C. Abood M. E. Alexander S. P. H. Di Marzo V. Elphick M. R. Greasley P. J. Hansen H. S. Kunos G. Mackie K. Mechoulam R. Ross R. A. (2010). International union of basic and clinical Pharmacology. LXXIX. Cannabinoid receptors and their ligands: beyond CB1and CB2. Pharmacol. Rev. 62 (4), 588–631. 10.1124/pr.110.003004 21079038 PMC2993256

[B57] Qiu J. Zhou S. Liu Q. (2016a). Energy metabolic alterations in the progression of atrial fibrillation: potential role of AMP-activated protein kinase as a critical regulator. Int. J. Cardiol. 212, 14–15. 10.1016/j.ijcard.2016.03.014 27015643

[B58] Qiu J. Zhou S. Liu Q. (2016b). Phosphorylated AMP-activated protein kinase slows down the atrial fibrillation progression by activating Connexin43. Int. J. Cardiol. 208, 56–57. 10.1016/j.ijcard.2016.01.201 26828380

[B60] Rajesh M. Batkai S. Kechrid M. Mukhopadhyay P. Lee W.-S. Horvath B. Holovac E. Cinar R. Liaudet L. Mackie K. Hasko G. Pacher P. (2012). Cannabinoid 1 receptor promotes cardiac dysfunction, oxidative stress, inflammation, and fibrosis in diabetic cardiomyopathy. Diabetes 61 (3), 716–727. 10.2337/db11-0477 22315315 PMC3282820

[B59] Rajesh M. Mukhopadhyay P. Haskó G. Liaudet L. Mackie K. Pacher P. (2010). Cannabinoid-1 receptor activation induces reactive oxygen species-dependent and -independent mitogen-activated protein kinase activation and cell death in human coronary artery endothelial cells. Br. J. Pharmacol. 160 (3), 688–700. 10.1111/j.1476-5381.2010.00712.x 20590572 PMC2931568

[B61] Ramirez F. D. Reddy V. Y. Viswanathan R. Hocini M. Jaïs P. (2020). Emerging technologies for pulmonary vein isolation. Circ. Res. 127 (1), 170–183. 10.1161/circresaha.120.316402 32716722

[B62] Schmid P. C. Schwartz K. D. Smith C. N. Krebsbach R. J. Berdyshev E. V. Schmid H. H. O. (2000). A sensitive endocannabinoid assay. The simultaneous analysis of N-acylethanolamines and 2-monoacylglycerols. Chem. Phys. Lipids 104 (2), 185–191. 10.1016/s0009-3084(99)00124-3 10669310

[B63] Severs N. J. Bruce A. F. Dupont E. Rothery S. (2008). Remodelling of gap junctions and connexin expression in diseased myocardium. Cardiovasc. Res. 80 (1), 9–19. 10.1093/cvr/cvn133 18519446 PMC2533424

[B65] St-Pierre J. Drori S. Uldry M. Silvaggi J. M. Rhee J. Jäger S. Handschin C. Zheng K. Lin J. Yang W. Simon D. K. Bachoo R. Spiegelman B. M. (2006). Suppression of reactive oxygen species and neurodegeneration by the PGC-1 transcriptional coactivators. Cell 127 (2), 397–408. 10.1016/j.cell.2006.09.024 17055439

[B64] Sterin-Borda L. Del Zar C. F. Borda E. (2005). Differential CB1 and CB2 cannabinoid receptor-inotropic response of rat isolated atria: endogenous signal transduction pathways. Biochem. Pharmacol. 69 (12), 1705–1713. 10.1016/j.bcp.2005.03.027 15885656

[B66] Sugamura K. Sugiyama S. Nozaki T. Matsuzawa Y. Izumiya Y. Miyata K. Nakayama M. Kaikita K. Obata T. Takeya M. Ogawa H. (2009). Activated endocannabinoid system in coronary artery disease and antiinflammatory effects of cannabinoid 1 receptor blockade on macrophages. Circulation 119 (1), 28–36. 10.1161/circulationaha.108.811992 19103987

[B67] Tiyerili V. Zimmer S. Jung S. Wassmann K. Naehle C. P. Lütjohann D. Zimmer A. Nickenig G. Wassmann S. (2010). CB1 receptor inhibition leads to decreased vascular AT1 receptor expression, inhibition of oxidative stress and improved endothelial function. Basic Res. Cardiol. 105 (4), 465–477. 10.1007/s00395-010-0090-7 20361197

[B68] Vara D. Salazar M. Olea-Herrero N. Guzmán M. Velasco G. Díaz-Laviada I. (2011). Anti-tumoral action of cannabinoids on hepatocellular carcinoma: role of AMPK-dependent activation of autophagy. Cell Death Differ 18 (7), 1099–1111. 10.1038/cdd.2011.32 21475304 PMC3131949

[B69] Ventura-Clapier R. Garnier A. Veksler V. (2008). Transcriptional control of mitochondrial biogenesis: the central role of PGC-1. Cardiovasc. Res. 79 (2), 208–217. 10.1093/cvr/cvn098 18430751

[B70] Wagner J. A. Hu K. Bauersachs J. Karcher J. Wiesler M. Goparaju S. K. Kunos G. Ertl G. (2001). Endogenous cannabinoids mediate hypotension after experimental myocardial infarction. J. Am. Coll. Cardiol. 38 (7), 2048–2054. 10.1016/s0735-1097(01)01671-0 11738314

[B71] Wakili R. Voigt N. Kääb S. Dobrev D. Nattel S. (2011). Recent advances in the molecular pathophysiology of atrial fibrillation. J. Clin. Invest. 121 (8), 2955–2968. 10.1172/jci46315 21804195 PMC3148739

[B72] Weresa J. Pędzińska-Betiuk A. Kossakowski R. Malinowska B. (2019). Cannabinoid CB1 and CB2 receptors antagonists AM251 and AM630 differentially modulate the chronotropic and inotropic effects of isoprenaline in isolated rat atria. Pharmacol. Rep. 71 (1), 82–89. 10.1016/j.pharep.2018.09.008 30500553

[B73] Wiersma M. Meijering R. A. M. Qi X. Y. Zhang D. Liu T. Hoogstra-Berends F. (2017). Endoplasmic reticulum stress is associated with autophagy and cardiomyocyte remodeling in experimental and human atrial fibrillation. J. Am. Heart Assoc. 6 (10), e006458. 10.1161/jaha.117.006458 29066441 PMC5721854

[B74] Wiersma M. van Marion D. M. S. Wüst R. C. I. Houtkooper R. H. Zhang D. Groot N. M. S. Henning fnm. Brundel fnm. (2019). Mitochondrial dysfunction underlies cardiomyocyte remodeling in experimental and clinical atrial fibrillation. Cells 8 (10), 1202. 10.3390/cells8101202 PMC6829298 31590355

[B75] Wijffels M. C. E. F. Kirchhof C. J. H. J. Dorland R. Allessie M. A. (1995). Atrial fibrillation begets atrial fibrillation. Circulation 92 (7), 1954–1968. 10.1161/01.cir.92.7.1954 7671380

[B76] Wolf P. A. Mitchell J. B. Baker C. S. Kannel W. B. D'Agostino R. B. (1998). Impact of atrial fibrillation on mortality, stroke, and medical costs. Arch. Intern. Med. 158 (3), 229–234. 10.1001/archinte.158.3.229 9472202

[B77] Woods A. Vertommen D. Neumann D. Türk R. Bayliss J. Schlattner U. Wallimann T. Carling D. Rider M. H. (2003). Identification of phosphorylation sites in AMP-activated protein kinase (AMPK) for upstream AMPK kinases and study of their roles by site-directed mutagenesis. J. Biol. Chem. 278 (31), 28434–28442. 10.1074/jbc.m303946200 12764152

[B78] Yan J. Kong W. Zhang Q. Beyer E. C. Walcott G. Fast V. G. Ai X. (2013). c-Jun N-terminal kinase activation contributes to reduced connexin43 and development of atrial arrhythmias. Cardiovasc. Res. 97 (3), 589–597. 10.1093/cvr/cvs366 23241357 PMC3567788

[B79] Yoshizawa T. Niwano S. Niwano H. Tamaki H. Nakamura H. Igarashi T. Oikawa J. Satoh A. Kishihara J. Murakami M. Fukaya H. Ako J. (2018). Antiremodeling effect of xanthine oxidase inhibition in a canine model of atrial fibrillation. Int. Heart J. 59 (5), 1077–1085. 10.1536/ihj.17-391 30158379

[B80] Zhang D. Hu X. Li J. Liu J. Baks-Te Bulte L. Wiersma M. (2019). DNA damage-induced PARP1 activation confers cardiomyocyte dysfunction through NAD(+) depletion in experimental atrial fibrillation. Nat. Commun. 10 (1), 1307. 10.1038/s41467-019-09014-2 30898999 PMC6428932

[B81] Zheng X. Sun T. Wang X. (2013). Activation of type 2 cannabinoid receptors (CB2R) promotes fatty acid oxidation through the SIRT1/PGC-1α pathway. Biochem. Biophysical Res. Commun. 436 (3), 377–381. 10.1016/j.bbrc.2013.05.108 23747418

[B82] Zhou L.-Y. Liu J.-P. Wang K. Gao J. Ding S.-L. Jiao J.-Q. Li P.-F. (2013). Mitochondrial function in cardiac hypertrophy. Int. J. Cardiol. 167 (4), 1118–1125. 10.1016/j.ijcard.2012.09.082 23044430

